# Speaking with a KN95 face mask: a within-subjects study on speaker adaptation and strategies to improve intelligibility

**DOI:** 10.1186/s41235-022-00423-4

**Published:** 2022-07-30

**Authors:** Sarah E. Gutz, Hannah P. Rowe, Victoria E. Tilton-Bolowsky, Jordan R. Green

**Affiliations:** 1 Program in Speech and Hearing Bioscience and Technology, Harvard Medical School, Boston, MA USA; 2 Department of Communication Sciences and Disorders, MGH Institute of Health Professions, Building 79/96, 2nd floor, 13th Street, Boston, MA 02129 USA

**Keywords:** Face masks, Speaker adaptation, Compensation, Speaker strategies

## Abstract

Mask-wearing during the COVID-19 pandemic has prompted a growing interest in the functional impact of masks on speech and communication. Prior work has shown that masks dampen sound, impede visual communication cues, and reduce intelligibility. However, more work is needed to understand how speakers change their speech while wearing a mask and to identify strategies to overcome the impact of wearing a mask. Data were collected from 19 healthy adults during a single in-person session. We investigated the effects of wearing a KN95 mask on speech intelligibility, as judged by two speech-language pathologists, examined speech kinematics and acoustics associated with mask-wearing, and explored KN95 acoustic filtering. We then considered the efficacy of three speaking strategies to improve speech intelligibility: Loud, Clear, and Slow speech. To inform speaker strategy recommendations, we related findings to self-reported speaker effort. Results indicated that healthy speakers could compensate for the presence of a mask and achieve normal speech intelligibility. Additionally, we showed that speaking loudly or clearly—and, to a lesser extent, slowly—improved speech intelligibility. However, using these strategies may require increased physical and cognitive effort and should be used only when necessary. These results can inform recommendations for speakers wearing masks, particularly those with communication disorders (e.g., dysarthria) who may struggle to adapt to a mask but can respond to explicit instructions. Such recommendations may further help non-native speakers and those communicating in a noisy environment or with listeners with hearing loss.

## Introduction

The COVID-19 pandemic has triggered a substantial increase in the use of face masks by the general public in the USA and worldwide due to mask mandates and recommendations from health organizations and governments (CDC, 2022; Fisher et al., 2020; OSHA, 2011). Moreover, masks have long been used in some workplace settings, such as hospitals and construction sites, and have been worn more habitually in some Asian countries to staunch the spread of disease.

### Impacts of mask-wearing on the acoustic speech signal

The extant literature in the field has reported that face masks can act as low-pass filters (Goldin et al., 2020; Saeidi et al., 2016) and attenuate overall intensity (Atcherson et al., 2017). Corey et al. (2020) reported that frequencies above 4 kHz are most affected in speech. The authors evaluated acoustic impacts for speakers wearing a mask, as well as for pre-recorded speech played through a mask. They found a peak intensity attenuation of 4 dB for KN95 respirators and surgical masks, 6 dB for N95 respirators, 4–12 dB for various cloth masks, and 8 dB for transparent masks (Corey et al., 2020). To put these findings in perspective, a 3 dB decrease equates to half the acoustic energy or 82% of perceptual loudness. Earlier work has found that oxygen masks can alter the transfer function of the vocal tract, resulting in distorted formants (Bond et al., 1989; Vojnovic et al., 2018), which are critical for speech sound recognition.

### Impacts of mask-wearing on intelligibility

Given the impacts of acoustic filtering on perceived loudness and speech recognition, a growing body of research has focused on how face coverings might affect intelligibility. Thus far, the effects of masks on speech intelligibility are mixed, with some studies showing no effect and others showing a mild or even significant speech intelligibility reduction. The difference in findings may be explained by variations in the mask types used, recording and listening conditions, and listeners. The impacts of masks on speech intelligibility and comprehensibility may also be due to reduced visual cues available to the listener (Fraser et al., 2010; Garcia et al., 2004; Ross et al., 2007). Indeed, previous work has found that removing visual information can degrade intelligibility when wearing a mask (Llamas et al., 2008). However, the impact of masks on intelligibility has varied across controlled studies that have isolated the acoustic impacts by playing pre-recorded speech through masks. Palmiero et al. (2016) reported 3–17% intelligibility loss for speech played through various N95 masks. Bottalico et al. (2020) found that surgical masks decreased the intelligibility of pre-recorded speech by 12%, N95 masks by 13%, and fabric masks by 16%. Toscano and Toscano (2021) tested the intelligibility of mask-wearing speakers, thereby implicitly including potential contributions from human compensation. They reported decreased intelligibility of masked speech only when speech was mixed with multi-talker babble (Toscano & Toscano, 2021). The authors also found little to no impact of surgical masks on speech recognition by human listeners and a decrease in recognition accuracy of 18.2% for cloth masks and 10% for N95 respirators. Radonovich et al. (2010) reported more varied, non-significant, intelligibility losses ranging from 1–17% for speakers wearing various N95 masks. Although not all work has reported intelligibility loss for speakers wearing surgical, N95, and cloth face masks (Magee et al., 2020), reductions in intelligibility may be more apparent for listeners with hearing impairments (Atcherson et al., 2017; Saunders et al., 2020). While the exact link between acoustic filtering and changes in intelligibility has not been established, an overall decrease in loudness may reduce the speech signal relative to the noise floor. Additionally, while most phonemic cues in typical speech are concentrated in the frequencies below 4 kHz (Ladefoged & Johnson, 2011; Monson et al., 2014), low-pass filters may impact the distinctiveness of phonemes characterized by high-frequency energy, such as fricatives and aspirated voiceless stops (Fecher & Watt, 2011; Ladefoged & Johnson, 2011).

### Speaker adaptations to mask-wearing

There has also been emerging evidence that speakers may compensate for the presence of a mask, particularly through voice changes. While one study found no changes in vocal quality when wearing a mask (Magee et al., 2020), self-report survey studies have found increased fatigue, vocal effort, and emotional stress after wearing or communicating with a mask for an extended period (McKenna et al., 2021; Ribeiro et al., 2020; Saunders et al., 2020). Some studies have demonstrated that mask-wearing speakers increase their vocal intensity (Asadi et al., 2020; Gutz et al., 2021). Changes in vocal load suggest phonatory compensation to the mask’s attenuation (Asadi et al., 2020), although it is unclear whether such compensation is in response to the overall attenuation or the attenuation of high frequencies only. Finally, since masks partially occlude the vocal tract, they could induce physiological voice changes in voice quality measures such as jitter and shimmer (Titze, 2006).

Moreover, increased fatigue when speaking with a mask could reflect other compensatory changes, such as increases in articulatory effort. Vowel formants provide critical cues for vowel perception (Stevens et al., 1969) and are strong indicators of speech intelligibility (Turner et al., 1995). Therefore, mask-wearers may respond by over- or “hyper-” articulating, a well-documented form of adaptation (Lindblom, 1990). Articulatory adaptations to improve speech clarity have been observed for other demanding speaking conditions, such as in noise (Darling & Huber, 2011), for hard-of-hearing listeners (Picheny et al., 1985), and following miscommunication (Buz et al., 2016). Indeed, in our previous work, we found increased vowel space area when people were wearing a mask (Gutz et al., 2021). However, it is not clear whether this increased articulatory distinctiveness was driven by changes to the first resonance of the vocal tract (i.e., F1), which tongue and jaw height influence, or by the second resonance (i.e., F2), associated with tongue advancement and retraction (Lee, 2014). Because wearing a mask could impede jaw movement, we may see temporary, adaptive changes similar to those seen in jaw or acoustic perturbation studies (Tremblay et al., 2003), such as decreased jaw movement and, as a result, reduced F1 range. Therefore, we might expect that changes in vowel space area could be primarily driven by increased F2 range.

Such adaptations in speech may have implications for researchers or clinicians collecting speech samples. Indeed, if wearing a mask triggers a substantial deviation from a habitual mode of speaking, then speech samples from masked individuals will not be valid representations of typical speech. Additionally, speech therapy may be less generalizable between masked and unmasked speech if mask-wearing prompts distinct speaking modes (Rochet-Capellan et al., 2012). Therefore, even recommendations to use amplification during data collection (Magee et al., 2020) may not be adequate if speech production with a mask deviates from typically produced speech.

### Impact of explicit speaking strategies on intelligibility and speech production

Given the detrimental impacts of mask-wearing on intelligibility, there is a need for empirical research into strategies masked speakers can employ to improve their intelligibility. Many strategies for intelligibility may be out of a speaker’s control, such as minimizing background noise in a public setting. Other methods rely on access to facial or gestural cues, which is not possible during voice calls or for many people with disabilities. Additionally, if masks reduce intelligibility and decrease the signal saliency by removing cues or lowering the signal-to-noise ratio, then any augmented cues would help bolster speech intelligibility. The American Speech-Language-Hearing Association (ASHA, 2021a) and Mheidly et al. (2020) both suggest several strategies for overcoming the impact of masks, including using supplemental gestures, exaggerating and attending to upper face expressions, and speaking slowly and loudly. For speaking mode strategies, a few recent studies have shown that Clear speech can improve masked speech intelligibility (Cohn et al., 2021; Yi et al., 2021). However, to our knowledge, the impact of other strategies, such as Loud and Slow speech, while wearing a mask has not yet been tested empirically.

All three strategies have been shown to increase speech intelligibility in individuals with motor speech disorders (Fox et al., 2006; Krause & Braida, 2002; Lam & Tjaden, 2013; Tjaden et al., 2014; Yi et al., 2021), although the results for Slow speech are not always favorable (Tjaden et al., 2014). In our prior work, Clear and Loud speech also improved ASR performance during mask-wearing, which suggests promise for improving intelligibility, although Slow speech did not improve ASR performance (Gutz et al., 2021). Acoustic and kinematic changes associated with these speaking modes have been well-reported. Prior work has noted increased articulator distinctiveness for Clear and Loud speech (Fox et al., 2006; Lam et al., 2012) and increased articulator excursion with corresponding increases in articulator speed, due to a greater travel distance for Clear, Loud, and Slow speech (Dromey & Ramig, 1998; Mefferd, 2017). Additionally, both Clear and Loud speech have been associated with increased speaker effort (Whitfield, et al., 2021). For masked speakers, we previously reported that the Clear condition resulted in a significantly larger vowel space area, and the Clear, Loud, and Slow conditions all resulted in significantly reduced speaking rate and increased intensity (Gutz et al., 2021).

Testing the efficacy of these strategies and expanding on current research is essential because feasibility and intelligibility could inform recommendations. Likewise, the cognitive effort required for a given strategy could have cascading consequences for the speaker and must also be considered (Kurzban et al., 2013).

### The current study

In this study, we recorded young, healthy adults reading sentences with and without a KN95 face mask. We subsequently tested the effects of three specific speaking strategies (i.e., Clear, Loud, and Slow speech) on speech produced while wearing a mask. We examined the impacts of wearing a mask in combination with implementing speaking strategies on speech performance using acoustic- and kinematic-based measures. Additionally, we evaluated sentence intelligibility to determine the functional impact of wearing a mask and using speaking strategies. Lastly, we explored perceived speaker effort for each condition to better understand how feasible it would be for speakers to use each strategy. We also calculated the acoustic filter of the KN95 mask to determine human compensation compared to the pure acoustic effects of the mask on voice measures.

#### Research questions

We sought to address the following research questions: (RQ1) How are individuals naturally adapting and changing their speech in response to wearing a mask? And (RQ2) What is the impact of explicit speaking strategies on intelligibility and speech production measures while wearing a mask?

We expected that speakers might adapt to the mask to improve intelligibility by directly compensating to the mask—i.e., countering the mask’s filter—and by exaggerating speech features that the mask’s filter does not directly impact—e.g., vowel distinctiveness and head movement to indicate paralinguistic cues. These predictions guided the measures we chose to investigate, as explained below.

## Methods

Table 1 includes a detailed summary of protocols and conditions. We previously reported results for speaking rate, speaking intensity, vowel space area, and automatic speech recognition (ASR) performance for this dataset (Gutz et al., 2021).

**Table 1:** Stimuli and measures for each protocol and condition

Protocol	Conditions	Stimulus	Outcome measure	
KN95 mask acoustic profile	Mannequin: “No Mask” “Mask”	Computer-generated white noise	Signal attenuation (Mask minus No Mask)	
			1/3 octave band analysis	
Human	Human: “No Mask” “Mask Only”	Sustained /a/	Phonatory measures (LHR, duration, F0, shimmer, jitter, HNR)	
	Human: “No Mask” “Mask Only” “Clear + Mask” “Loud + Mask” “Slow + Mask”	VAS survey	Speaker effort	
		SIT	Transcription intelligibility	
		Story read task	Formant measures (F1 and F2 range)	
		Spoken paragraph	Kinematic measures (Jaw ROM and speed; Head ROM and Speed)	
Phonatory compensation to KN95 mask	Human + mannequin: “Masked Human” (maskless mannequin) “Masked Mannequin” (maskless human)	Human-produced sustained /a/ from “No Mask” and “Mask Only” conditions	Phonatory measures (LHR, duration, F0, shimmer, jitter, HNR)	

### Protocol: human speakers (see Table 1)

#### Participants: speakers

Speakers were 19 individuals (14 female, five male; *M* = 26.7 years, *SD* = 4.3, *range* = 20–36) who spoke North American English as their native language. Participants had no reported history of speech, language, or neurological impairment, and they reported normal vision and hearing. Participants were recruited through a weekly email sent to hospital employees and volunteers to recruit healthy participants.

#### Participants: raters

Two speech-language pathologists (SLPs), one with seven years’ and one with 18 years’ experience evaluating and treating adults with speech and language disorders, provided perceptual judgments of samples produced by speakers during the Sentence Intelligibility Test (SIT, Yorkston, Beukelman, & Hakel, 2007; described below).

#### Speaker protocol

Participants first completed all tasks with instructions to speak in their normal speaking voices, once with a KN95 mask (herein referred to as the “Mask Only” condition) and once without a mask (“No Mask” condition), in a counterbalanced order across participants. Participants then completed the Clear + Mask, Loud + Mask, and Slow + Mask conditions wearing a KN95 mask, in a counterbalanced order (“Clear,” “Loud,” and “Slow” conditions, respectively). A brief pre-experiment practice of at least three Harvard sentences (Rothauser et al., 1969) preceded the Clear, Loud, and Slow conditions, with a spoken model and general feedback provided by the investigator. After each condition, participants rated their effort using a paper VAS scale (see below) and partook in casual conversation for 2–3 min to wash out any effects of the speech instructions from the preceding condition. Participants produced sustained /a/ and read a series of sentences in each condition, which are described in Table 1 and expanded upon below.

Speakers completed all tasks in a separate room to maintain a safe physical distance during the protocol, and they communicated with the experimenter via video and audio call. The experimenter provided speakers with real-time feedback to ensure that individual performance within conditions was similar. Instructions for each condition were based on previous research investigating effective speaking mode instructions:

- *No Mask and Mask Only:* “Speak in your normal speaking voice.”
- *Clear* + *Mask:* “Speak clearly, making sure you overenunciate each word. If your regular speech corresponds to a clearness of 100, you should aim for a clearness twice as good or a clearness of 200” (Lam et al., 2012; Tjaden et al., 2013).
- *Loud* + *Mask:* “Speak loudly. If your regular speech corresponds to a loudness of 100, you should speak twice as loudly, or at a loudness of 200” (Tjaden et al., 2013).
- *Slow* + *Mask:* “Speak slowly. If your regular speech corresponds to a rate of 100, speak at a rate half as fast, corresponding to a rate of 50.” Participants were further encouraged to stretch out speech sounds, rather than inserting pauses (Tjaden et al., 2013).

#### Measures

Both SLP raters were blinded to condition, and speakers were not told the expected outcomes of the study. When comparing the No Mask and Mask Only conditions, our primary measures of interest were kinematic and acoustic mechanisms of speech performance, which allowed us to quantify adaptation. We also considered speech intelligibility in these conditions as a means of relating speech changes to their functional impact.

The primary outcome measure during the speaking strategy conditions was intelligibility, as this measure provides insight into the strategies’ functional impact. However, we also considered their kinematic and acoustic effects to (1) better understand how these strategies interact with the presence of the mask and (2) provide preliminary insight into which clinical populations may be best able to implement each strategy.

#### Functional impact: transcription intelligibility

All speakers completed the Sentence Intelligibility Test (SIT; Yorkston, Beukelman, & Hakel, 2007), which consists of 11 sentences that increase incrementally in length from five to 15 words. For each SIT set, the sentences were chosen randomly from a set of 1089 sentences to minimize repetitions of sentences that any listener may hear. Each SIT set was unique to each participant–condition combination, and stimuli were hand-checked to ensure that no speaker read the same sentence twice across conditions. SLP raters were not familiar with the sentences beforehand. We presented only the four longest SIT sentences (12–15 words in length) to listeners, as longer sentences have been found to be more sensitive to intelligibility changes (Allison et al., 2019). Two SLP listeners transcribed the sentences over two sessions and were allowed to take breaks as needed. Prior work on sentence intelligibility has demonstrated high intra- and inter-rater reliability for two raters (Stipancic et al., 2016). There were 380 total samples (19 speakers * five conditions * four sentences) ordered randomly across speakers and conditions. Each listener judged half of the sentences (190 samples). We also included 38 intra-rater reliability sentences (20% of samples) and 38 inter-rater reliability sentences (20% of samples, 10% chosen from each listener’s set). Thus, each listener transcribed 247 total sentences. Sentences were presented one at a time, and listeners were permitted to listen to each sentence no more than twice.

We collected SLP-provided transcription intelligibility remotely through an online survey platform, REDCap (Harris et al., 2019). SIT sentences were mixed with multi-talker babble (Healy et al., 2013) to reduce a potential ceiling effect, as per (Lam & Tjaden, 2013). The intensity level of the babble for each speaker was calibrated for each speaker to achieve a signal-to-noise ratio (SNR) of -1 dB in the habitual condition, an SNR chosen based on our prior work in ASR recognition of the same speech recordings (Gutz et al., 2021). Furthermore, we used the same absolute level of noise for all of a given speaker’s productions to ensure that speaker-produced intensity changes would result in an increase of the speech signal over the noise, as they would in a real-life situation. Each sample was normalized between -1 and 1 to ensure a comfortable and consistent listening volume across productions. While this normalization changed the relative intensity of each sample, any advantage of speaking louder would be maintained through the increased SNR over the multi-talker babble. The listeners wore headphones while completing the task and were presented with a training sample so that they could adjust their headphone volume to a comfortable volume before beginning the task.

SLP-provided transcription intelligibility for each sentence was calculated using the Python jiwer library, which compares two strings and calculates a word error rate (Vaessen, 2020). We subtracted this word error rate from 1 and multiplied it by 100 to obtain percent intelligibility. Unlike traditional hand scoring, this automatic method penalizes transcriptions that have additional words inserted into the transcription and cannot account for typos or homophones. However, high agreement between the computer and human scoring (presented below) indicated that computer scoring resulted in minimal to no change in intelligibility scores. Overall transcription intelligibility was computed as the percentage of the total number of target words across all sentences that were correctly transcribed for a given speaker and condition.

#### Functional impact: speaker effort

Immediately after each condition, speakers rated their perceived effort on an unmarked paper 100 mm visual analog scale. Ratings were then converted to a scale of 0 to 1, where 1 corresponded to higher effort.

#### Mechanism of change: acoustic measures

Measures related to voice and low/high ratio were extracted from a sustained /a/ elicited from speakers during the No Mask and the Mask Only conditions. Low/high ratio was calculated as the ratio of energy present in frequency bands below 4 kHz to energy present in frequency bands above 4 kHz, following Corey et al. (2020) and Lowell and Hylkema (2016). Intensity was collected using a calibrated sound pressure level meter (A-weighting). Phonatory measures were calculated automatically from audio recordings of sustained /a/ using a customized Praat script. Phonatory measures that may have been impacted by a semi-occluded vocal tract were collected, including sustained /a/ duration, F0, shimmer, jitter, and harmonic-to-noise ratio (HNR). HNR was calculated using Praat’s autocorrelation method, as described in Boersma (1993), and as used in previous work to determine the relative periodicity of the signal (e.g., Brockmann-Bauser, et al., 2018).

Formant measures were taken from corner vowels /i, æ, u, a/ produced in a within-sentence, /bXt/ or /bXb/ context as part of a story read task (Green et al., 2010). Vowels were hand segmented and formant settings were verified for each sample by a single judge who was blinded to condition. Formant extraction was performed using a Praat (Boersma & Weenink, 2006) script that extracted the mean F1 and F2 from the linear predictive coding spectrum of the middle 30 ms of each vowel (Hustad et al., 2010; Tjaden & Wilding, 2004). F1 range was calculated for each participant and condition as the absolute difference between mean F1 values for high vowels, /i/ and /u/, and mean F1 values for low vowels, /æ/ and /a/ (Lam et al., 2012). F2 range was calculated for each participant and condition as the absolute difference between mean F2 values for front vowels, /i/ and /æ/, and mean F2 values for back vowels, /a/ and /u/ (Lam et al., 2012). Both F1 and F2 ranges are acoustic measures of vowel distinctiveness. F1 range is strongly influenced by both tongue and jaw movement (Lee, 2014) and measures the acoustic distinctiveness of high and low vowels. F2 range mainly reflects tongue advancement and retraction (Lee, 2014) and measures the acoustic distinctiveness of front and back vowels.

#### Mechanism of change: kinematic measures

Electromagnetic articulography data were collected using Wave (NDI) with one six degrees of freedom (DOF) sensor on the forehead, one five DOF sensor on the chin, and one six DOF sensor on the jugular notch of the manubrium. The sternal sensor was used for reference when tracking forehead movement, and the forehead sensor was subtracted from the chin sensor in order to calculate independent jaw movement. Analyses were conducted using SMASH, a customized MATLAB program (Green et al., 2013). To ensure safe social distancing, speakers applied the 3D electromagnetic sensors themselves, which the experimenter verified via a real-time video feed.

Range of motion (ROM) was calculated as the volume of the ellipsoid (mm^3^) created by the movement of each sensor over a spoken paragraph (Yunusova et al., 2016). As in previous studies, measurements more than 2.5 standard deviations from the mean were excluded from this calculation (Yunusova et al., 2016). Jaw and head movement speed (mm/s) were likewise calculated over this passage by computing the first derivative of the 3D Euclidean distance time series.

#### Statistical measures

We used mixed-effects models with condition (Mask Only, No Mask, Clear, Loud, Slow) as the predictor and participant as a random effect to examine the effect of these factors on the dependent measures under investigation. We used the lmerTest package in R and the equation lmer(measure ~ condition + [1|participant]) (Kuznetsova et al., 2017). For each measure, we also investigated the impact of sex by including it as a fixed effect and interaction: lmer(measure ~ condition * sex + [1|participant]), with female as the reference. However, we only report results by sex for measures that differed significantly between the sexes. Furthermore, since we found no interactions between sex and condition, it was appropriate to combine data from the sexes because the random intercept of participant would account for individual differences.

Because both research questions could be addressed by comparing conditions to the Mask Only condition, the Mask Only condition was used as the reference condition in all analyses. Due to our small sample size, we report both significance levels and effect sizes (standardized beta coefficients, abbreviated as Beta or *B* throughout), as effect sizes are better indicators of group differences in small samples (Gaeta & Brydges, 2020; Sullivan & Feinn, 2012). As per recommendations for speech research in Gaeta and Brydges (2020), an effect size of 0.25 was interpreted as a small effect, 0.55 as a medium effect, and 0.95 as a large effect (Cohen, 1988).

### Protocol: mannequin and KN95 mask (see Table 1)

In a sound-attenuating booth, an acoustic signal was played through a speaker (Scanspeak Discovery 5F/8422 T-01 2" Full Range) embedded in a polystyrene foam mannequin head and recorded (32-bit mono, 44.1 kHz) using a head-fixed microphone 5.1 cm from the mannequin mouth.

#### Mask acoustic profile

To measure the acoustic filter of the mask, computer-generated white noise was played through the mannequin both while it was and was not wearing a KN95 mask.

#### Phonatory compensation to the mask

To determine the effects of human compensation independent of the mask’s acoustic filtering, we played human-produced sustained /a/ in two conditions. In one condition, the original recordings were produced by a mask-wearing human and played through the mannequin without a mask (“Masked Human”). In the second condition, the original recordings were produced by a maskless human and played through a mask-wearing mannequin (“Masked Mannequin”). In both conditions, the speech signal was recorded through the same mask, microphone, and speaker. The two conditions varied only in terms of whether the human or the mannequin wore the mask, that is, whether the human had an opportunity to compensate for the mask.

We tested the impact of human compensation only on voice measures in order to disentangle the contributions of the mask’s filter and human phonatory compensation. Previous work has already established that the mask’s filter impacts intelligibility (Bottalico et al., 2020) and we would not expect the mask’s filter to directly impact other measures such as vowel space or speaking rate.

#### Stimuli and measures

**Fig. 1:**
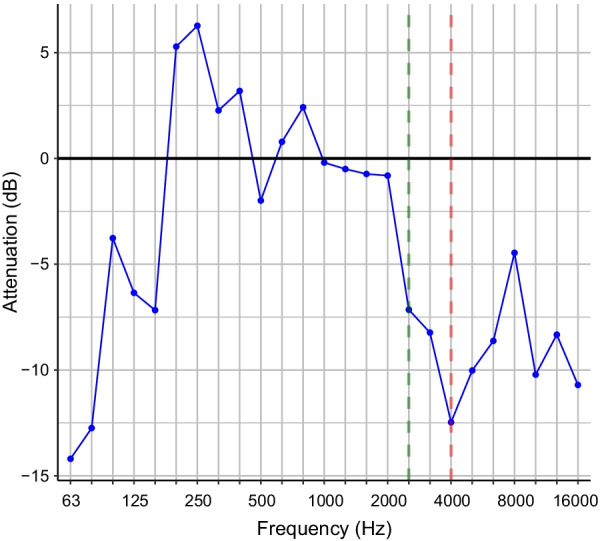
KN95 mask attenuation of white noise—1/3 octave band analysis. *Note:* Intensity attenuation of KN95 mask on white noise, presented by 1/3 octave bands. Negative values indicate lower intensity when played through the mask compared to noise not played through the mask. Red dashed line = low/high-frequency cutoff A; Green dashed line = low/high-frequency cutoff B

*Acoustic filtering of mask* We performed fast Fourier transforms on recordings of white noise that were played through the mannequin head. The signals were converted to dB (A-weighted) relative to the noise floor recorded in the sound booth, and we performed 1/3 octave band analysis for bands 63 Hz—16 kHz, similar to Bottalico et al. (2020). The signal of the noise recorded without a mask (in dB) was subtracted from the signal of the noise recorded with a mask at all frequencies to determine the impact of acoustic filtering. We compared the average attenuation for frequency ranges 80 Hz—16 kHz (full spectrum), 80 Hz—4 kHz (low frequencies A), and 4—16 kHz (high frequencies A) based on previous work on mask filtering (Bottalico et al., 2020; Corey et al., 2020). We also computed average attenuation for ranges with cutoffs at 2.5 kHz, the frequency above which the mask always attenuated the signal by at least 2.5 dB (low and high frequencies B, see Fig. 1). Similar to Bottalico et al. (2020), we performed 1/3 octave band analysis on the signal for bands with center frequencies 63 Hz—16 kHz. We present descriptive analyses of these data.

#### Phonatory measures

Phonatory measures were calculated from sustained /a/ in an identical manner to the purely human protocol described above. These measures were low/high ratio, sustained /a/ duration, F0, shimmer, jitter, and harmonic-to-noise ratio (HNR).

#### Statistical measures

As for the human protocol, we used mixed-effects models with condition (Masked Human, Masked Mannequin) as the predictor and participant as a random effect to examine the effect of these factors on the dependent measures under investigation. As above, we used the lmerTest package in R as lmer(measure ~ condition + [1|participant]).

## Results

Since there was not an interaction between sex and condition for any measures, we pooled the data across sexes, particularly given that analyses were within-subject. We report results by sex only for measures that differed by sex: F0, F1 range, and F2 range.

### Reliability

For acoustic measures, the same analyst re-measured 10% of the speech samples, and intraclass correlation coefficients (ICCs) were computed to determine intra-rater reliability (Stipancic et al., 2016). Because acoustic measures were computed algorithmically, variations in measures would be due to differences in parsing. Analyses revealed that variation in parsing had little to no effect as indicated by an ICC = 0.949, *F*(9,8.59) = 44.1 for F1 range; ICC = 0.979, *F*(9,9.96) = 95.6 for F2 range; and ICC = 1 for all phonatory measures derived from sustained /a/ (*p* < 0.001 for all), all well above the acceptable range for ICC (Koo & Li, 2016).

For SIT transcription, 20% of samples overlapped between the two listeners to assess inter-rater reliability. In addition, each listener rated 20% of samples twice to assess intra-rater reliability. Analyses revealed good inter-rater reliability as indicated by an ICC = 0.849, *F*(16,16.7) = 11.9, *p* < 0.001. Moderate intra-rater reliability was found for SLP 1 with an ICC = 0.716, *F*(14,15) = 6.15, *p* < 0.001 and good intra-rater reliability for SLP 2 with an ICC = 0.802, *F*(14,11.8) = 10.6, *p* < 0.001.

To assess the reliability of automatic intelligibility scoring for perceptual analyses, a blinded scorer hand-scored 10% of all SIT transcriptions. Excellent reliability was found between human and computer scoring of SIT transcriptions; ICC = 0.987, *F*(49, 43.5) = 163, *p* < 0.001.

Reliability for kinematic measures was not tested as they were computed fully algorithmically.

### KN95 mask acoustic profile

Many of the results for the KN95 mask acoustic profile are descriptive. The Mask attenuated the signal over the entire frequency range, and especially in higher frequencies above 4 kHz and above 2.5 kHz (see Table 2). The mask added a resonance from 178 to 269 Hz, with boundaries at zero-crossings, with an average gain of 9.35 dB and a peak gain at 258 Hz (see Fig. 1).

**Table 2:** KN95 acoustic profile

Spectrum section	Lower frequency	Upper frequency	Intensity difference	
			Mean (dB)	*SD* (dB)
Full spectrum	80 Hz	16 kHz	− 7.47	4.60
Low frequencies A	80 Hz	4 kHz	− 4.16	5.91
High frequencies A	4 kHz	16 kHz	− 8.56	3.45
Low frequencies B	80 Hz	2.5 kHz	− 0.36	3.35
High frequencies B	2.5 kHz	16 kHz	− 8.76	3.48
Mask resonance	178 Hz	269 Hz	+ 9.35	3.73

Average intensity difference between the mask and no mask conditions for white noise played through the mannequin speaker, calculated as mask minus no mask. Lower and upper frequency indicates the boundaries of each spectrum section

### Functional impact

#### Transcription intelligibility

**Fig. 2:**
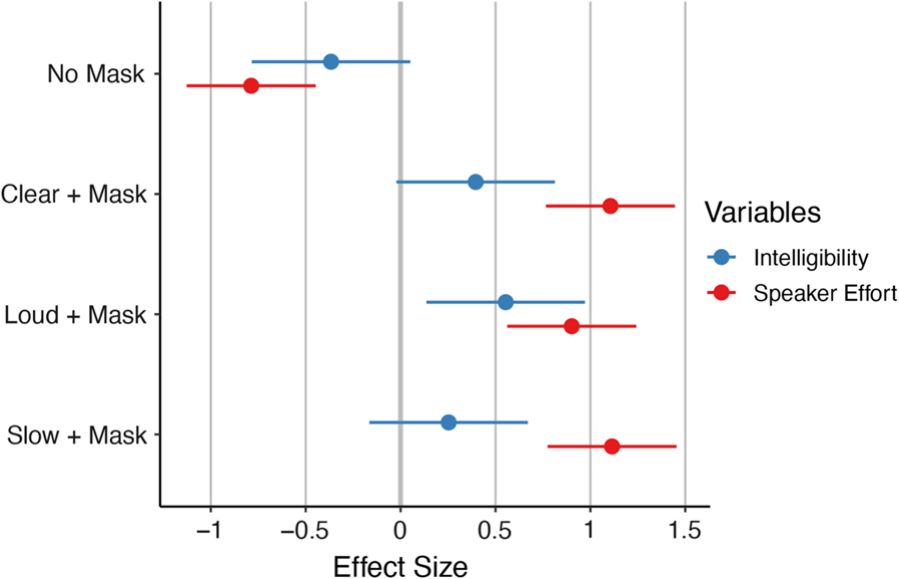
Effect sizes of functional measures relative to mask only. *Note:* Standardized beta coefficients for transcription intelligibility (blue) and speaker effort (red) for each condition relative to the Mask Only condition. Negative effect size indicates lower values relative to the Mask Only condition; positive effect size indicates higher values relative to the Mask Only condition. Error bars indicate 95% confidence interval. Large effect: |Beta|≥ 0.95; medium effect: |Beta|≥ 0.55; small effect: |Beta|≥ 0.25

Speech intelligibility in the Mask Only condition did not significantly differ from the No Mask condition *B* = -0.37, *SE* = 0.21*, t*(72) = -1.72*, p* = 0.090, although there was a small increase in intelligibility for the Mask Only condition. The Loud condition resulted in significantly more intelligible speech than the Mask Only condition *B* = 0.55, *SE* = 0.21*, t*(72) = 2.60*, p* = 0.011. The Clear and Slow conditions also resulted in higher intelligibility, although these had non-significant, small effect sizes, of *B* = 0.40, *SE* = 0.21*, t*(72) = 1.86*, p* = 0.067 for Clear and *B* = 0.25, *SE* = 0.21*, t*(72) = 1.19*, p* = 0.238 for Slow. There was no significant effect of sex *B* = -0.67, *SE* = 0.38, *t*(17) = -1.78, *p* = 0.090 (see Table 3 and Fig. 2).

**Table 3:** Functional impact—intelligibility and effort for human speakers

	Intelligibility (%)		Speaker effort	
	Mean	*SD*	Mean	*SD*
Mask only	89.38	12.39	0.36	0.27
No mask	84.86	14.84	0.09	0.16
Clear + Mask	94.25	12.87	0.74	0.21
Loud + Mask	96.20	5.26	0.67	0.24
Slow + Mask	92.50	11.91	0.74	0.27

Descriptive statistics mean and standard deviation (*SD*) for functional measures of transcription intelligibility and speaker effort

#### Speaker effort

Self-rated effort in the Mask Only condition was significantly greater than in the No Mask condition *B* = -0.79, *SE* = 0.17, *t*(72) = -4.54, *p* < *0.001*. Additionally, effort was greater than the Mask Only condition for the Clear condition, *B* = 1.11, *SE* = 0.17, *t*(72) = 6.38, *p* < *0.001*; Loud condition *B* = 0.90, *SE* = 0.17, *t*(72) = 5.20, *p* < *0.001*; and Slow condition, *B* = 1.11, *SE* = 0.17, *t*(72) = 6.43, *p* < *0.001*. There was no significant effect of sex, *B* = 0.19, *SE* = 0.25, *t*(17) = 0.79, *p* = 0.440 (see Table 3 and Fig. 2).

### Mechanism of change: acoustic measures

#### Phonatory measures: human protocol

**Fig. 3:**
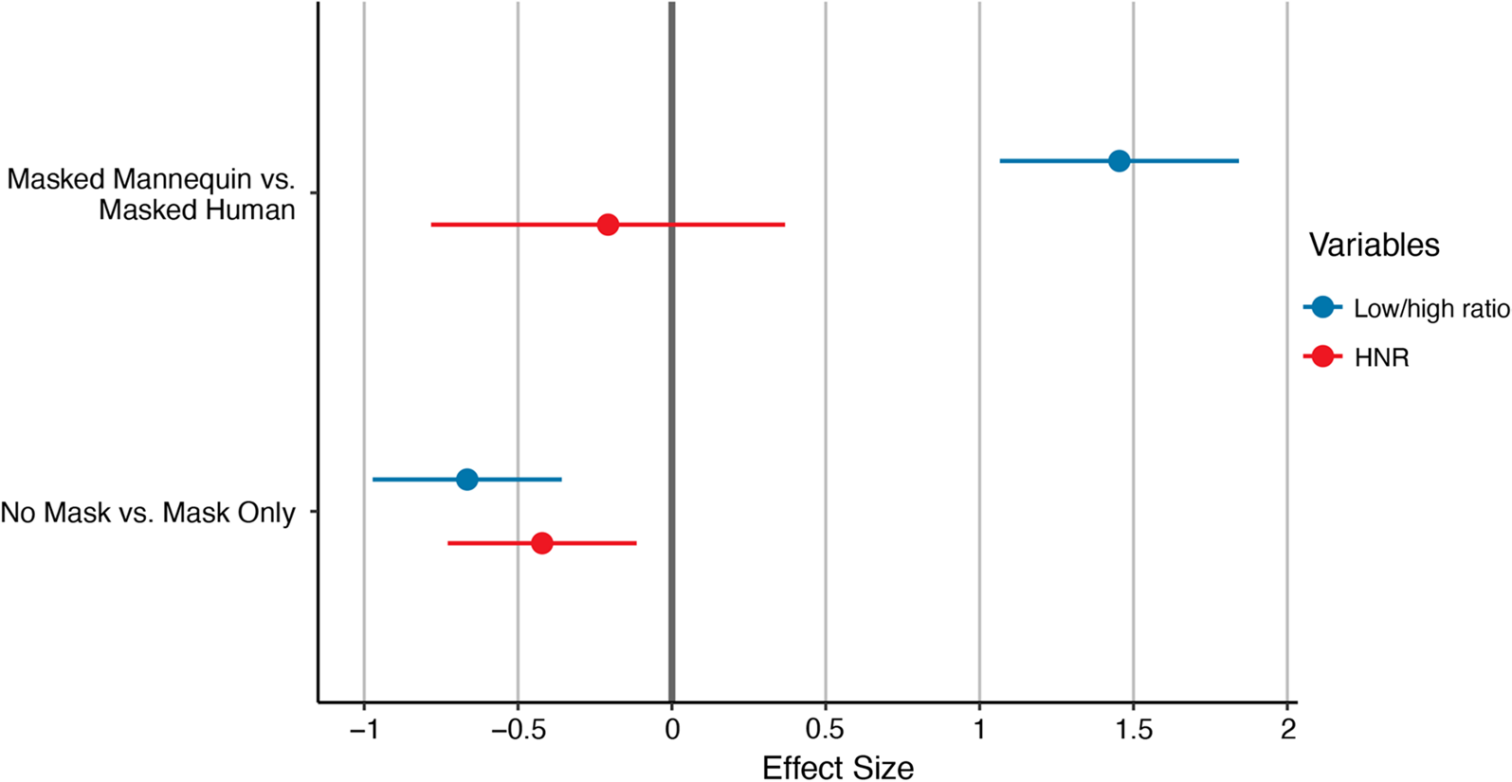
Effect size of select phonatory measures. *Note:* Standardized beta coefficients for low/high ratio (blue) and harmonic-to-noise ratio (HNR, red) of Masked Mannequin relative to Masked Human (top) and human-produced No Mask relative to Mask Only (bottom). Negative effect size indicates lower values relative to the reference condition; positive effect size indicates higher values relative to the reference condition. Error bars indicate 95% confidence interval. Large effect: |Beta|≥ 0.95; medium effect: |Beta|≥ 0.55; small effect: |Beta|≥ 0.25

Low/high ratio was significantly higher in the Mask Only condition relative to the No Mask condition, *B* = *-0.67, SE* = *0.16, t(18)* = *-4.24, p* < *0.001,* indicating a relative concentration of energy in lower frequencies (80 Hz—4 kHz) compared to higher frequencies (4 -10 kHz). Energy in the lower frequencies (80 Hz—4 kHz) was not significantly different between the No Mask and Mask Only conditions, *B* = -0.09, *SE* = 0.12, *t(*18) = -0.80, *p* = 0.434. There was significantly more high-frequency energy (4 -10 kHz) in the No Mask condition than the Mask Only condition *B* = 0.48, *SE* = 0.11, *t*(18) = 4.26, *p* < 0.001. There was no significant effect of sex on low/high ratio, *B* = 0.27, *SE* = 0.47, *t*(17) = 0.56, *p* = 0.580; low-frequency energy, *B* = 0.46, *SE* = 0.51, *t*(17) = 0.90, *p* = 0.379; or high-frequency energy, *B* = 0.13, *SE* = 0.51, *t*(17) = 0.25, *p* = 0.802 (see Table 4 and Fig. 3).

**Table 4:** Mechanism of change—phonatory measures for human speakers

	Mask only		No mask	
	Mean	*SD*	Mean	*SD*
Low frequencies (dB)	77.20	5.07	76.82	4.04
High frequencies (dB)	36.24	5.53	38.78	4.82
Low/high ratio (dB)	40.96	4.39	38.04	4.82
Duration (s)	19.63	9.32	20.33	9.78
F0 (Hz)	189.60	43.93	184.32	45.24
Shimmer (%)	0.04	0.01	0.04	0.01
Jitter (%)	0.004	0.001	0.004	0.002
HNR (dB)	22.26	3.14	21.01	2.70

	Female speakers		Male speakers	
F0 (Hz)	209.30	24.51	124.39	16.71

Descriptive statistics mean and standard deviation (*SD*) for phonatory measures produced by humans. Low frequencies: 80 Hz–4 kHz; High frequencies: 4–10 kHz

Between the Mask Only and No Mask conditions, there was no difference in sustained /a/ duration, *B* = 0.74, *SE* = 0.12*, t*(18) = 0.59*, p* = 0.561; F0, *B* = -0.12, *SE* = 0.10*, t*(18) = -1.23*, p* = 0.234; shimmer, *B* = -0.05, *SE* = 0.20*, t*(18) = -0.24*, p* = 0.813; or jitter *B* = 0.15, *SE* = 0.20*, t*(18) = 0.75*, p* = 0.462. Harmonic-to-noise ratio (HNR) was significantly higher in the Mask Only condition when compared to the No Mask condition, *B* = -0.42, *SE* = 0.16*, t*(18) = -2.69*, p* = 0.015.

F0 was significantly lower for males than for females, *B* = -1.93, *SE* = 0.25*, t*(17) = -7.68*, p* < 0.001. There were no other significant differences between sexes (see Table 4 and Fig. 3).

#### Phonatory measures: masked human versus masked mannequin

Low/high ratio was significantly higher in the Masked Mannequin condition when compared to the Masked Human condition, *B* = 1.45, *SE* = 0.20*, t*(18) = 7.34*, p* < 0.001 (see Table 5 and Fig. 3). Energy in the low frequencies (80 Hz—4 kHz) was significantly lower in the Masked Mannequin condition compared to the Masked Human condition, *B* = -0.43, *SE* = 0.14, *t*(18) = -3.01, *p* = 0.007. There was also significantly less high-frequency energy (4 -10 kHz) in the Masked Mannequin condition than in the Masked Human condition, *B* = -1.41, *SE* = 0.14, *t*(18) = -10.33, *p* < 0.001.

**Table 5:** Mechanism of change—phonatory measures played through a mannequin

	Masked Human		Masked Mannequin	
	Mean	*SD*	Mean	*SD*
Low frequencies (dB)	76.23	4.13	74.26	4.90
High frequencies (dB)	42.23	6.76	30.74	4.55
Low/high ratio (dB)	34.00	4.62	43.52	4.35
Duration (s)	19.63	9.32	20.33	9.78
F0 (Hz)	189.26	43.73	185.10	45.13
Shimmer (%)	0.04	0.01	0.04	0.02
Jitter (%)	0.006	0.001	0.005	0.004
HNR (dB)	21.69	4.29	20.92	3.13

Descriptive statistics mean and standard deviation (*SD*) for phonatory measures produced by humans and played through the mannequin. Low frequencies: 80 Hz–4 kHz; high frequencies: 4–10 kHz

Between the Masked Mannequin and Masked Human conditions there were no differences in sustained /a/ duration, *B* = 0.08, *SE* = 0.13*, t*(18) = 0.60*, p* = 0.556; F0, *B* = -0.09, *SE* = 0.09*, t*(18) = -1.05*, p* = 0.309; shimmer, *B* = 0.11, *SE* = 0.26*, t*(18) = 0.42*, p* = 0.678; jitter, *B* = -0.14, *SE* = 0.33*, t*(18) = -0.42*, p* = 0.680; or HNR, *B* = -0.21, *SE* = 0.30*, t*(18) = -0.71*, p* = 0.489 (see Table 5 and Fig. 3).

#### F1 range

**Fig. 4:**
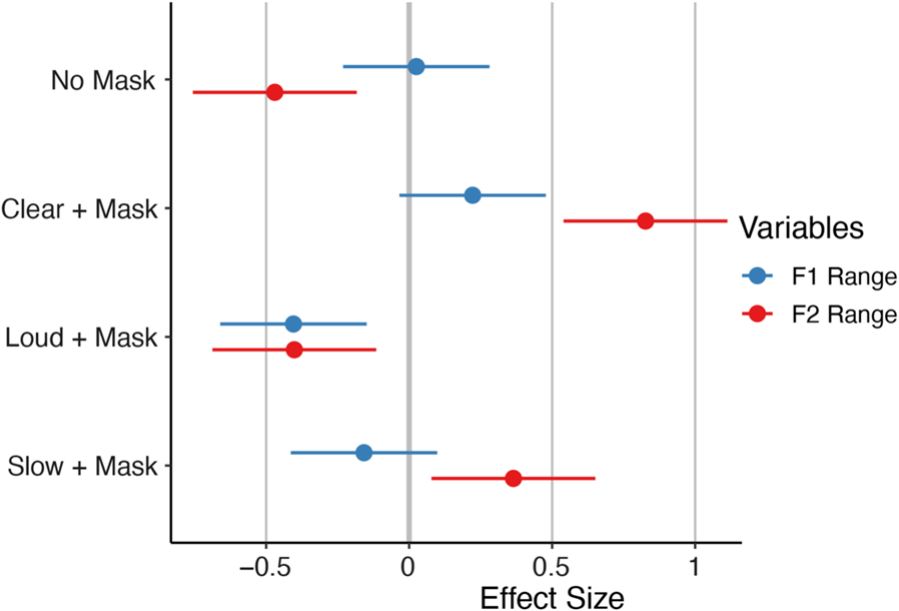
Effect sizes of formant measures relative to mask only. *Note:* Standardized beta coefficients for F1 range (blue) and F2 range (red) for each condition relative to the Mask Only condition. Negative effect size indicates lower values relative to the Mask Only condition; positive effect size indicates higher values relative to the Mask Only condition. Error bars indicate 95% confidence interval. Large effect: |Beta|≥ 0.95; medium effect: |Beta|≥ 0.55; small effect: |Beta|≥ 0.25

F1 range was significantly smaller in the Loud condition compared to the Mask Only condition, *B* = -0.40, *SE* = 0.13*, t*(167) = -3.10*, p* = 0.002. There was no difference in F1 range comparing the Mask Only to the No Mask condition, *B* = 0.03, *SE* = 0.13*, t*(167) = 0.19*, p* = 0.847; Clear condition, *B* = 0.22, *SE* = 0.13*, t*(167) = 1.70*, p* = 0.091; or Slow condition, *B* = -0.16, *SE* = 0.13*, t*(167) = -1.21*, p* = 0.229. Further, we found a significant effect of sex on F1 range, in that F1 range was lower for males than females, *B* = -1.28, *SE* = 0.33*, t*(17) = -3.94*, p* = 0.001. There was no interaction between sex and condition, *p* > 0.05 for all conditions (see Table 6 and Fig. 4).

**Table 6:** Mechanism of change—formant measures for human speakers

	F1 range (Hz)		F2 range (Hz)	
	Mean	*SD*	Mean	*SD*
Mask only	177.24	83.78	490.10	262.61
No mask	179.73	88.62	350.73	162.61
Clear + Mask	199.22	119.40	735.30	346.12
Loud + Mask	137.19	79.96	371.03	224.35
Slow + Mask	161.61	109.90	598.28	274.15
Female	204.23	90.58	566.30	305.32
Male	77.95	48.42	348.90	194.01

Descriptive statistics mean and standard deviation (*SD*) for F1 range and F2 range

#### F2 range

The Mask Only condition had a significantly larger F2 range than the No Mask condition, *B* = -0.47, *SE* = 0.15*, t*(167) = -3.22*, p* = 0.002. Compared to the Mask Only condition, F2 range was significantly larger in the Clear condition, *B* = 0.83, *SE* = 0.15*, t*(167) = 5.66*, p* < 0.001, and the Slow condition, *B* = 0.36, *SE* = 0.15*, t*(167) = 2.50*, p* = 0.014. The Loud condition produced a significantly smaller F2 range than the Mask Only condition, *B* = -0.40, *SE* = 0.15*, t*(167) = -2.75*, p* = 0.007. Males had a significantly smaller F2 range than females, *B* = -0.73, *SE* = 0.30*, t*(17) = -2.46*, p* = 0.025, but there was no interaction between sex and condition, *p* > 0.05 for all conditions (see Table 6 and Fig. 4).

### Mechanism of change: kinematic measures

#### Jaw ROM

**Fig. 5:**
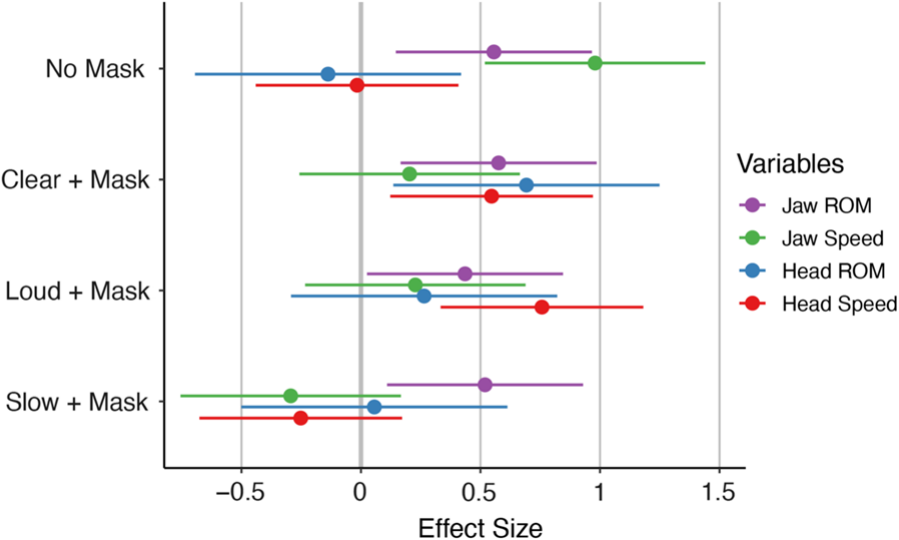
Effect sizes of kinematic measures relative to mask only. Note. Standardized beta coefficients for Jaw ROM (purple), jaw speed (green), head ROM (blue), and head speed (red) for each condition relative to the Mask Only condition. Negative effect size indicates lower values relative to the Mask Only condition; positive effect size indicates higher values relative to the Mask Only condition. Error bars indicate 95% confidence interval. Large effect: |Beta|≥ 0.95; medium effect: |Beta|≥ 0.55; small effect: |Beta|≥ 0.25

Jaw ROM was smallest in the Mask Only condition and was significantly smaller than the ROM in the No Mask condition, *B* = 0.56, *SE* = 0.21*, t*(72) = 2.66*, p* = 0.010. Jaw ROM was significantly larger than the Mask Only condition in the Clear condition, *B* = 0.58, *SE* = 0.21*, t*(72) = 2.75*, p* = 0.007; Loud condition *B* = 0.44, *SE* = 0.21*, t*(72) = 2.08*, p* = 0.041; and Slow condition, *B* = 0.52, *SE* = 0.21*, t*(72) = 2.48*, p* = 0.015. This effect was largest in the Clear condition. Sex had no impact on Jaw ROM, *B* = -0.44, *SE* = 0.42*, t*(17) = -1.03*, p* = 0.318 (see Table 7 and Fig. 5).

**Table 7:** Mechanism of change—kinematic measures for human speakers

	Jaw ROM (mm^3^)		Jaw speed (mm/s)	
	Mean	*SD*	Mean	*SD*
Mask only	93.80	104.05	32.36	12.76
No mask	220.80	157.35	50.96	28.99
Clear + Mask	225.31	210.69	36.23	15.81
Loud + Mask	193.24	245.32	36.68	11.96
Slow + Mask	212.36	345.86	26.78	12.17

	Head ROM (mm^3^)		Head speed (mm/s)	
Mask only	1662.99	2447.11	23.20	6.15
No mask	1140.15	1276.60	23.07	6.26
Clear + Mask	4291.61	6638.28	27.61	9.67
Loud + Mask	2666.58	3675.84	29.31	8.05
Slow + Mask	1875.98	1937.34	21.17	7.43

Descriptive statistics mean and standard deviation (*SD*) for kinematic measures for the jaw (top) and head (bottom)

#### Jaw speed

Jaw speed was significantly faster in the No Mask condition than in the Mask Only condition, *B* = 0.98, *SE* = 0.24*, t*(72) = 4.17*, p* < 0.001. Jaw speed was slower in the Slow condition compared to the Mask Only condition, *B* = -0.29, *SE* = 0.24*, t*(72) = -1.25*, p* < 0.337; this was a small, non-significant difference. There was no difference between the Mask Only condition and the Clear condition, *B* = 0.21, *SE* = 0.24*, t*(72) = 0.87*, p* = 0.390, or the Loud condition, *B* = 0.23, *SE* = 0.24*, t*(72) = 0.97*, p* = 0.337. And there was no difference between the sexes, *B* = -0.32, *SE* = 0.35*, t*(17) = -0.92*, p* = 0.371 (see Table 7 and Fig. 5).

#### Head ROM

Head ROM was significantly larger in the Clear condition than in the Mask Only condition, *B* = 0.69, *SE* = 0.28*, t*(72) = 2.44*, p* = 0.010. Head ROM was also larger in the Loud condition, *B* = 0.26, *SE* = 0.28*, t*(72) = *0.* 93*, p* = 0.355, though this was a small, non-significant effect. There was no difference in change in head ROM between the Mask Only condition and the Slow condition, *B* = 0.06, *SE* = 0.28*, t*(72) = 0*.*20*, p* = 0.844, or the No Mask condition, *B* = -0.14, *SE* = 0.28*, t*(72) = -0.49*, p* = 0.629. Sex had no significant impact on head ROM, *B* = -0.27, *SE* = 0.31*, t*(17) = -0.87*, p* = 0.394 (see Table 7 and Fig. 5).

#### Head speed

Relative to the Mask Only condition, head speed was significantly faster in the Clear condition, *B* = 0.55, *SE* = 0.22*, t*(72) = 2.53*, p* = 0.014, and the Loud condition,, *B* = 0.76, *SE* = 0.22*, t*(72) = 3.50*, p* < 0.001. Head speed was slower in the Slow condition, *B* = -0.25, *SE* = 0.22*, t*(72) = -1.16*, p* = 0.248; this was a small, non-significant effect. There was no difference in head speed between the Mask Only and No Mask conditions, *B* = -0.02, *SE* = 0.22*, t*(72) = -0.08*, p* = 0.941; or between the sexes, *B* = -0.09, *SE* = 0.39*, t*(17) = -0.24*, p* = 0.815 (see Table 7 and Fig. 5).

## Discussion

### Summary of findings

Overall, our findings suggest that speakers are adapting their articulatory patterns when wearing a mask. These adaptations appear to overcome losses in both intensity and intelligibility caused by the mask, which has been similarly reported in studies that isolated the acoustic impact of masks on intelligibility by playing recordings of maskless speech through masks (Bottalico et al., 2020; Palmiero et al., 2016). Moreover, speaking loudly or clearly improved intelligibility while wearing a mask, but speaking slowly did not have the same positive effect. Finally, speakers reported that speaking with a mask required more effort than speaking without a mask, and the additional task of implementing speech strategies required more effort than wearing a mask without using explicit strategies. These findings have implications for people wearing a mask who are looking to improve their intelligibility, as well as researchers and clinicians who work with mask-wearing speakers.

## Mask only

### Functional impact

#### Preserved intelligibility when wearing mask

Although the mask significantly attenuated high frequencies, intelligibility was generally preserved, and possibly slightly improved, as we found a non-significant, small effect of increased intelligibility when wearing a mask (see Fig. 2). This finding is consistent with our recent observation that automatic speech recognition (ASR) was unaffected by masks (Gutz et al., 2021) and some previous work showing limited effects on listeners with normal hearing (Atcherson et al., 2017). These findings are somewhat at variance with prior work showing decreased intelligibility from mask-wearing (Atcherson et al., 2017; Bottalico et al., 2020; Llamas et al., 2008; Palmiero et al., 2016). However, whereas some of the aforementioned work played pre-recorded speech through masks (Bottalico et al., 2020; Palmiero et al., 2016), we assessed the intelligibility of speech produced while speakers were wearing a mask, thereby including the effects of speaker compensation. Our results are, therefore, more in line with studies that also recorded speakers wearing masks and which did not find statistically significant intelligibility decrements in controlled recording environments (Magee et al., 2020; Radonovich et al., 2010; Toscano & Toscano, 2021). Toscano and Toscano (2021) did find reduced intelligibility for cloth and N95 masks, but only when the authors mixed the speech with multi-talker babble at an SNR of + 3 dB. In contrast to this study, we mixed speech with multi-talker babble at an SNR of -1 dB, collected intelligibility judgments from highly trained SLP listeners, and tested KN95 masks, all of which could impact intelligibility results. Furthermore, our methodology differs from Llamas et al. (2008), who tested cloth face coverings and surgical masks for just two speakers, and from Atcherson et al. (2017) who only tested one speaker and found that masks impacted intelligibility primarily for listeners with hearing loss. The design features of our study allowed us to examine underlying speaker adaptations to mask-wearing rather than the effects of competing noise or untrained listeners.

### Mechanism of change

#### Compensation to filtering effect of mask

At the acoustic level, the results from both our human protocol and acoustic profile protocol were consistent with prior work showing the significant low-pass filtering properties of masks. For human speakers, the Mask Only condition had an increased low/high ratio relative to the No Mask condition, indicating a higher concentration of low frequencies in the signal when people were phonating with a mask. We also noted substantially greater attenuation of high-frequency energy in our acoustic analysis of the KN95 mask filter.

In addition to the acoustic impact of the mask, we found evidence that human speakers were at least partially compensating for the mask’s low-pass filter. When we compared the Masked Mannequin and Masked Human conditions, we observed a greater low/high ratio in the Masked Mannequin condition relative to the Masked Human condition. These results suggest that masked humans were actively boosting the relative intensity of high-frequency components in their speech signal to counteract the low-pass filter.

Furthermore, both low- and high-frequency components were lower in the Masked Mannequin condition than in the Masked Human condition. These results suggest that the Masked humans were compensating by increasing their intensity across the spectrum, in addition to increasing the relative intensity of high-frequency components.

#### Decreased loudness due to mask, but potentially increased vocal effort

Additionally, we measured an average decrease in intensity of 7.47 dB for the pure acoustic impact of the mask; this finding was greater than for prior work on KN95 masks, which found a 4 dB decrease (Corey et al., 2020). In our previously reported work on this data, however, we did not find a significant decrease in speaking intensity for the Mask Only condition compared to the No Mask condition (Gutz et al., 2021), and in the current study, we found increased energy across the spectrum for the Masked Human. Such maintenance of vocal intensity during mask-wearing suggests behavioral adaptation and aligns with previous work that found increased vocal effort and spectral tilt with mask-wearing (McKenna et al., 2021). Under unmasked speaking conditions, vocal intensity can be increased through respiratory, phonatory, and articulatory mechanisms that increase vocal source intensity or enhance sound radiation efficiency (Zhang, 2016). The increased speaker-reported effort in the Mask Only condition may be reflecting an increase in physiologic effort required to increase intensity overall and high-frequency energy (Zhang, 2016).

We also observed an increase in HNR for the Mask Only condition, which is consistent with previous work (Nguyen et al., 2021). Similar to vocal loudness, an increased HNR has been associated with increased vocal intensity and vocal effort (McKenna et al., 2021). However, a more plausible explanation based on our findings is that the mask blocks high-frequency noise while letting lower frequency harmonics pass, thereby increasing the HNR. This explanation aligns with our finding of no difference in HNR between the Masked Human and Masked Mannequin conditions, indicating that changes in HNR were not due to human compensation.

#### Increased jaw and tongue movement when wearing mask

The formant measures provide further evidence for articulatory compensation during the Mask Only condition. Specifically, the increased F2 range in the context of attenuated jaw movements during the Mask Only condition indicates that speakers increased their tongue movements. In contrast, F1 was unchanged despite the expectation that it would decrease as a function of decreased jaw motion (Lindblom & Sundberg, 1970). These findings suggest that speakers’ responses to the degrading effects of the Mask on speech are similar to those elicited by speaking in noise (i.e., Lombard effect), which also induces over-articulation (Darling & Huber, 2011; Garnier et al., 2018; Zollinger & Brumm, 2011). Prior work has found that speakers adapt to consistent, repeated acoustic perturbations (Raharjo et al., 2021), and speakers revert to their habitual way of speaking once the perturbation is removed (Tremblay et al., 2003). We would, therefore, expect people to revert to their typical speech upon removal of a mask in response to the changing sensorimotor feedback. Thus, it does not seem likely that mask-induced articulatory changes will extend to unmasked speech.

#### Implications for data collection and speech therapy

Many of the differences we observed between the No Mask and Mask Only conditions could not be attributed to the mere acoustic impact of the mask; they indicated active speaker compensation in both acoustic and kinematic domains. Speech produced while wearing a mask is not typical either in the mechanics of production (e.g., jaw restriction) or functional results (e.g., intelligibility), and therefore, the presence of a mask may confound data collected either for clinic or for research. For data collection in either clinical or research settings, audio samples collected from a mask-wearing speaker may not reflect typical speech. Therefore, it could be difficult to disentangle the influences of a speaker’s habitual patterns, the tested experimental condition, and the mask.

For therapy implementation, literature on the principles of motor learning has demonstrated that practiced motor skills, such as speech, transfer most easily to more similar contexts (Rochet-Capellan et al., 2012). If wearing a mask prompts significant compensation, then there may be limited generalizability of practiced speech strategies between masked and unmasked conditions.

## Impact of speaking strategies

### Functional impact

#### Greatest intelligibility gains with loud and clear speech

As previously reported, speakers successfully adhered to instructions to change their speech in the Clear, Loud, and Slow conditions while wearing a mask (Gutz et al., 2021). We found increased intelligibility for speakers in each of these conditions. The largest increases in intelligibility were a medium-sized significant change in the Loud condition, followed by a non-significant small change in the Clear condition, which is consistent with previous work on these speaking strategies. We noted the smallest change in the Slow condition. Indeed, while most prior work involves speakers with speech impairment who are not wearing masks, evidence suggests that Loud and Clear speech improve intelligibility (Krause & Braida, 2002; Neel, 2009; Park et al., 2016; Tjaden et al., 2014; Wenke et al., 2008; Yi et al., 2021), whereas the effects of Slow speech on intelligibility are less favorable (Tjaden et al., 2014; ), even when used in conjunction with Clear speech (Krause & Braida, 2002). Clear speech has been found effective for clinical populations with dysarthria (Park et al., 2016), people with extensive public speaking experience (Krause & Braida, 2002), and neurologically typical adults without documented public speaking experience (Lam & Tjaden, 2013). Moreover, using the same data, we previously found that Clear speech was most effective for improving ASR performance, followed by Loud speech; Slow speech did not improve ASR accuracy (Gutz et al., 2021). However, the slight increase in intelligibility for the Mask Only condition we observed may be statistically minimizing potential intelligibility gains from each speaking strategy.

Loud speech may be most effective in noisy environments, where increasing speaking intensity can raise the speech signal relative to environmental noise. The power of Clear speech may be its flexibility, as the instructions allow speakers to rely on existing internal feedback mechanisms and find their own best strategy for speaking clearly. While speaking clearly and loudly can successfully improve intelligibility, these strategies may require additional vigilance and effort. Indeed, the self-reported level of effort in the Clear, Loud, and Slow conditions was significantly higher than that reported in the Mask Only condition.

Even though we observed a slow speaking rate in the Loud and Clear conditions (Gutz et al., 2021), results suggest that slowed speech is not an effective strategy, especially given the large increase in speaker effort it requires. Rather, slowed rate may be an effective mechanism or by-product of achieving Clear or Loud speech goals. Prior research has shown that slow speech can degrade speech motor performance by disrupting interarticulatory coordination (Toma et al., 2002; van Lieshout, 2017), or by decreasing the smoothness (Park et al., 2017) and spatiotemporal stability of articulator movements (Mefferd & Green, 2010). To that end, one possible reason the Loud condition produced the greatest increase in intelligibility is that, of the three strategies, it had the smallest decrease in speaking rate.

### Mechanism of change

#### Increased jaw and tongue movement with clear and slow strategies

Both Clear and Slow speech elicited larger jaw movements and F2 ranges, while Loud speech was primarily characterized by increased jaw movement that had little to no impact on the spectral properties of speech. Findings that both Clear and Slow speech prompted exaggerated tongue advancement and retraction (F2 range) and larger jaw movement (Jaw ROM) are in line with previous literature on the kinematics of Clear speech (Dromey, 2000; Hadar, 1991; Mefferd, 2017; Mefferd & Green, 2010). Particularly for the Slow condition, any necessary increase in jaw speed due to the increased ROM was likely offset by the overall reduced speaking rate in this condition (Gutz et al., 2021).

Intelligibility gains in the Loud condition may be primarily due to increased intensity rather than enhanced articulation. Significantly increased Jaw ROM is consistent with the association between Loud speech and larger articulator movements (Dromey & Ramig, 1998; Mefferd, 2017), although a decrease in F1 range is surprising given the connection between jaw height and F1 (Lindblom & Sundberg, 1970). These findings suggest that speakers may have increased their jaw movement for non-speech breaths or consonants (e.g., aspirated /p/) rather than for vowels. Indeed, while some work has found that Loud speech increases F1 range (Fox et al., 2006), other work has found no change in formant range during Loud speech (Koenig & Fuchs, 2019; Whitfield et al., 2018). It is possible that speakers in the Loud condition reverted to their habitual F1 and F2 ranges; such a change to normalcy could appear, statistically, as a decrease relative to the exaggerated F1 and F2 ranges in the Mask Only condition.

#### Increased head movement with clear and loud strategies

Changes in head kinematics for the Clear and Loud conditions suggest global, non-speech changes in the communication signal. Larger or more frequent head movements, such as nodding, can be used to emphasize syntactic boundaries and stress markers in speech (Hadar, 1991; McClave, 2000; Munhall et al., 2004; Wagner et al., 2014).

### Recommendations for speakers

Since masks decrease the saliency of acoustic and visual cues in the speech signal, communicators may be able to counteract these effects either by reducing background noise or by augmenting their communicative signal with speech changes or nonverbal cues (e.g., hand gestures or facial expressions) (Chodosh et al., 2020; Mattys et al., 2012). Based on our findings, speakers may benefit from employing Loud or Clear speech. Slow speech had a smaller impact on speech intelligibility, no impact on ASR accuracy (Gutz et al., 2021), and has a considerably worse track record for improving intelligibility.

Although we found positive effects from speaking strategies for improving intelligibility while wearing a mask, these strategies required increased effort. Speaking effort was higher when participants were wearing a mask, and higher still when they were employing speech strategies, especially for the Clear and Slow conditions.

Moreover, the Loud and Clear conditions both produced increased speech intensity (Gutz et al., 2021), and the unaltered intensity in the Mask Only condition suggests that speakers were increasing vocal drive while wearing a mask (McKenna et al., 2021). Increased vocal intensity carries a risk for vocal hyperfunction and potential vocal fold damage, especially when used habitually (van Stan et al., 2020), and may increase aerosol emissions (Schiff, 1990). Indeed, recent work has linked mask usage to increased reports of vocal fatigue (McKenna et al., 2021). Speakers who must speak for long periods with a mask, such as teachers or attorneys, could benefit from wearing voice amplifiers, which have been found to be effective with masks (Corey et al., 2020; Miller, 2013). While a microphone would not address the problem of the mask’s low-pass filter, it would help raise the signal above the noise floor (Miller, 2013). We further recommend that people who speak while wearing a mask for prolonged periods, regardless of whether they *consciously* increase their vocal intensity, refer to previous work on maintaining vocal health (e.g., ASHA, 2021b; Behrman et al., 2008; Diaz, 2020).

Given that reduced speech intensity is the most consistently reported impact of face masks and that speaking loudly with a mask improves intelligibility, reducing environmental noise could greatly benefit communication (Bradley et al., 2002). To that end, we recommend lowering or turning off music or television in places where people need to communicate, such as in stores and restaurants. Reducing background noise could minimize the need for mask-wearers to increase their volume, which, in turn, would lessen their effort expenditure. Speakers could, additionally, opt for environments that better facilitate communication, such as quiet public spaces, outdoor areas where they do not need to wear a mask, or spaces with improved room acoustics (Bottalico et al., 2016). Listeners with hearing loss (Atcherson et al., 2017) may also benefit more when speakers implement the recommended strategies or when the environment is optimized for audible communication.

Given the trade-offs between improving intelligibility and minimizing expended effort and vocal load, **speakers may benefit most from speaking loudly or clearly to boost their intelligibility only as needed** when it is apparent that their communication partners are mishearing or misinterpreting their verbal messages (e.g., for keywords, to repair miscommunications, or in a public setting where they have little control over environmental noise).

### Limitations and future directions

#### Sample size

Our study had several limitations. First, we collected data from a small set of speakers, and our sample of 19 participants was disproportionately female, with only four male participants. While we found well-established sex differences for F0, F1 range, and F2 range (Whiteside, 2001), we did not find any interactions between sex and condition.

#### Sample characteristics

Moreover, our speakers were all young adults and did not report any communication or cognitive impairments. The restrictions we placed on the participant sample allowed us to control for extraneous factors such as age-related vocal changes. Additionally, our young, healthy speakers were able to automatically compensate in the Mask Only condition, which may have statistically diminished the effect of the speaking strategies. Speakers who are incapable of adapting to the mask (e.g., due to neurologic or anatomic impairments) may see greater benefits from speaking strategies.

Our ability to generalize our findings to non-native speakers and speakers with impaired communication systems (e.g., dysarthria, aphasia, hearing loss) is limited by our sample population. However, given evidence that speaking strategies like Clear speech can benefit these people both as speakers and as listeners (Bradlow & Alexander, 2007; Cooke & Lecumberri, 2012; Fox et al., 2006; Lam & Tjaden, 2013; Tjaden et al., 2014; Yi et al., 2021; Yorkston et al., 2007a, 2007b), future work on mask-wearing and possible compensatory strategies is warranted in this area.

#### Ecological validity

We focused solely on the acoustic signal for perceptual analyses. Future work could use video recordings to investigate the impact of an impoverished visual communication signal and compare that to the impact of a pure acoustic effect, similar to Hustad and Cahill (2003). Furthermore, because speech samples were elicited in an experimental environment, speakers may have altered their speech or performed at their “best behavior.” While these conditions allowed us to control the stimuli and recording conditions, they may have lessened any adverse impacts of the mask on speech intelligibility. It is unclear if speakers would employ similar compensation (e.g., increasing F2 range) while wearing a mask in more ecological conditions.

We selected reading tasks to control the stimuli and speech targets. While speakers were connected to the experimenter via a video call throughout data collection, participants may have compensated more if they were engaged in a task-oriented paradigm with a true communicative goal, such as a map task in which speakers must communicate to reproduce a map route only one person can see (Thompson et al., 1993).

Finally, future studies could examine the impact of other mask types (Yi et al., 2021) as well as optimal mask type or strategy for specific speakers. Furthermore, because speakers reported increased effort while speaking with a mask, additional work could examine the relationship of effort with speech fatigue associated with wearing a mask (Ribeiro et al., 2020), and the effects of mask-wearing on cognitive load and performance (Adler & Benbunan-Fich, 2012; Cutsem et al., 2017).

## Conclusion

This study investigated the impacts of mask-wearing on speech and provided insight into the mechanisms of change underlying these impacts. We found that while the KN95 mask acted as a low-pass filter and restricted jaw movement, speakers adapted their speech through over-articulation (as indexed by increased formant range), increased vocal drive (as indexed by compensated speaking intensity), and increased high-frequency energy in their speech (as indexed by low/high ratio). Consequently, masked speakers improved their intelligibility beyond their unmasked speech. Instructions to speak clearly or loudly—and, to a lesser extent, slowly—further improved speech intelligibility while wearing a mask. These improvements, however, came at a cost, as speakers reported increased effort when using the compensatory strategies and while speaking with a mask. Therefore, we recommend that masked speakers use Loud or Clear speech only in situations where a decrease in their intelligibility is apparent, in order to communicate effectively without overexerting themselves.

## Data Availability

The datasets generated and analyzed during the current study are not publicly available due to participant privacy concerns and IRB restrictions.

## Abbreviations

ASHA: The American Speech-Language-Hearing Association
ASR: Automatic speech recognition
Beta: Standardized beta coefficient, an effect size measurement
DOF: Degrees of freedom
HNR: Harmonic-to-noise ratio
ICC: Intraclass correlation coefficient
ROM: Range of motion
SIT: Sentence Intelligibility Test
SLP: Speech-language pathologist
SNR: Signal-to-noise ratio
